# Analysis of Mobile Edge Computing for Vehicular Networks †

**DOI:** 10.3390/s19061303

**Published:** 2019-03-15

**Authors:** Zachary W. Lamb, Dharma P. Agrawal

**Affiliations:** Center for Distributed and Mobile Computing, EECS Department, University of Cincinnati, P.O. Box 210030, Cincinnati, OH 45221-0030, USA; agrawadp@ucmail.uc.edu

**Keywords:** cloud computing, distributed computing, mobile computing, VANET, wireless networks

## Abstract

Vehicular ad-hoc Networks (VANETs) are an integral part of intelligent transportation systems (ITS) that facilitate communications between vehicles and the internet. More recently, VANET communications research has strayed from the antiquated DSRC standard and favored more modern cellular technologies, such as fifth generation (5G). The ability of cellular networks to serve highly mobile devices combined with the drastically increased capacity of 5G, would enable VANETs to accommodate large numbers of vehicles and support range of applications. The addition of thousands of new connected devices not only stresses the cellular networks, but also the computational and storage requirements supporting the applications and software of these devices. Autonomous vehicles, with numerous on-board sensors, are expected to generate large amounts of data that must be transmitted and processed. Realistically, on-board computing and storage resources of the vehicle cannot be expected to handle all data that will be generated over the vehicles lifetime. Cloud computing will be an essential technology in VANETs and will support the majority of computation and long-term data storage. However, the networking overhead and latency associated with remote cloud resources could prove detrimental to overall network performance. Edge computing seeks to reduce the overhead by placing computational resources nearer to the end users of the network. The geographical diversity and varied hardware configurations of resource in a edge-enabled network would require careful management to ensure efficient resource utilization. In this paper, we introduce an architecture which evaluates available resources in real-time and makes allocations to the most logical and feasible resource. We evaluate our approach mathematically with the use of a multi-criteria decision analysis algorithm and validate our results with experiments using a test-bed of cloud resources. Results demonstrate that an algorithmic ranking of physical resources matches very closely with experimental results and provides a means of delegating tasks to the best available resource.

## 1. Introduction

Advancement in autonomous vehicle technologies has created a demand for higher-bandwidth, improved availability, and ubiquitous networking technologies. The aging capabilities of the dedicated short range communication (DSRC) standard, which were originally defined for vehicular communications, are being phased out in favor of modern and capable technologies. The pursuit of 5G next-generation networking technologies continues to push development of more robust and ubiquitous wireless communications. Bolstered by such advanced technologies, extensive deployment of vehicular ad-hoc networks (VANETs), capable of supporting a wide array of applications will soon be feasible. Traditional VANETs are ad-hoc in nature, meaning vehicles can communicate with one another without the need for any infrastructure. However, it is widely accepted that an effective vehicular network would require some form of static infrastructure such as roadside-units (RSUs), cell towers, or base stations. An infrastructure built on 5G would support such a network architecture since they are expected to operate in a cellular fashion, not unlike current cellular networks.

Recently, research has begun to explore the concept of cellular vehicular networks, referred to as cellular-Vehicle to Everything (C-V2X) [1,2,3,4]. In C-V2X, vehicles do not need to communicate directly with one another. Instead data-transfer takes place just as a cellular phone network, where base stations, or cell towers, cover an area and provide service to all the users in the cell. Communication between vehicles is facilitated by the cell tower and the message would pass from the source vehicle to tower and finally to the destination vehicle. In this scheme, vehicles only need to maintain contact with the cell tower, whereas pure vehicle-to-vehicle (V2V) would require a vehicle to negotiate communication with many other vehicles within its vicinity. However, this approach would require the use of the already stressed cellular infrastructure or the addition of new cell towers. Substantial progress has been made to an implementation of cellular V2X in the Long-Term Evolution (LTE) variant LTE-V that employs network functions virtualization (NFV). With NFV, the functions of LTE, such as routing, are virtualized and can be run on commoditiy hardware. This removes the vendor-specific hardware requirements of LTE and results in a much more flexible system. The benefits of LTE over the 802.11 p based DSRC standard include much higher capacity and greater communication range. An increased communication range is particularly valuable in vehicular networks, since this would allow any emergency information to be disseminated throughout the network more quickly. In terms of incident reporting, faster information dissemination could provide a safer driving environment.

In response to the progress towards 5G, researchers have begun exploring methods to incorporate the technology into the vehicular network domain. One proposed method of leveraging 5G and cellular technologies in vehicular communications is to introduce shared spectrum platform. In [5] the authors present an approach similar to works in which a combination of LTE and DSRC is proposed and seeks to create a shared spectrum combination of DSRC and 5G. While this may be further off than a combination of DSRC and LTE, coexistence of DSRC, cellular, and 5G would allow for a more robust scalable network that leverages both licensed and un-licensed bands. Sharing spectra, such as those defined for cellular and mmWave allows for full utilization of the available spectrum. Not only would this approach achieve a better utilization of the spectrum, it would allow new technologies to be rolled out alongside existing hardware and allow for an incremental transition to 5G. Continuing the pursuit of full spectrum utilization, additional works have explored the use of wavelengths in the free space optical (FSO) portion of the spectrum. The authors in [6] present experiments that show a simple infrared light emitting diode can suffice for optical communication with a mobile vehicle. In [7] a method for car-to-car, or V2V, is proposed and also uses simple light emitting diodes paired with a PIN photodiode at the receiver. While commodity hardware is more feasible, another approach would be to employ more powerful hardware to operate in a cellular fashion. For example, optical receivers could reside on the cell towers, or RSUs, and act at the relay for communications between vehicles. This would also allow the full bandwidth and throughput of FSO communication to be exploited when uploading data from vehicle to the roadside infrastructure. As noted in [8] an additional benefit of FSO is low power usage and immunity to RF interference. FSO also has some inherent security since the transmission beam will be very narrow and any intrusion would be immediately detected as a drop in signal strength. Immunity to RF interference makes FSO a great candidate for a shared spectrum technology and could perform well alongside existing RF communications. Employing FSO in vehicular communications presents another source of unlicensed spectrum as well as another means for high-capacity wireless communications. With many challenges involved in tracking, alignment, and attenuation, FSO has been explored only for simple applications such as intra-vehicle and V2V communication via LED light sources already available on most modern vehicles.

In any case, meeting the communication demands of vehicular networks necessitates a network architecture that can support highly mobile devices while maintaining the ability to handle both real-time messages and delay-tolerant data off-loads. As we progress toward semi, and eventually fully autonomous vehicles, demand for vehicle-collected data will increase greatly. The numerous on-board sensors, cameras, and LiDar systems are expected to generate Terabytes of data daily for each vehicle [9]. This data will be invaluable to both vehicle manufactures and researchers and can be leveraged to monitor and improve vehicle performance, relieve traffic congestion, and so on. However, accommodating this influx of new data will put additional strain on both networking and computing infrastructure.

Addressing the growing demand of future applications has also prompted researchers to explore edge-computing in vehicular networks. With future vehicles expected to possess a reasonable amount of computing resources, researchers have begun to explore the use of vehicles themselves as edge nodes in a distributed cloud system. In [10] the authors present a scheme that employs groups of vehicles as “micro-clouds” and can be used to aggregate and pre-process data before it is transferred to back-end resources. Clustering vehicles and electing a cluster-head (CH), allows for the data of multiple vehicles to be aggregated, which can reduce the amount of data communication between the access point, or RSU and reduce overall network traffic by transmitting fewer bytes. Other works have followed similar trends in which they employ computational and storage enabled RSUs to explore some of the hardware requirements for data-gathering in VANETs [11]. In this scenario, RSUs make an event-based decision on which data should be uploaded to the intelligent transportation system (ITS) servers. RSUs acting as a compute layer allows for decisions about which data to collect or reject and are made much closer to the network edge, thus reducing the load on the ITS servers and minimizing the amount of data that is transmitted through the network. Edge nodes with computational and storage capabilities also provide a means for efficient content delivery in vehicular networks [12]. Given sufficient storage space at edge nodes, the network has the potential to cache the contents and ensure a timely delivery to end users. This has added value for any location-based service that provides data to vehicles. Any information relevant to a particular location can be cached at a nearby Base Station and, made readily available to vehicles. This technique could also be extended to include file sharing between vehicles, assuming sufficient storage space is available on the vehicles hardware.

Incorporating a new source of data also has a significant impact on the computational and storage requirements. While it is safe to assume that an autonomous vehicle will process a large amount of its data in real-time, it is not unrealistic to assume that additional data must be transferred to remote locations for storage and later processing. Moving and processing data is costly and requires careful planning to minimize resource impact. In an environment as demanding as vehicular networks, the utilization of resources must be carefully managed. In traditional cloud computing, tasks and associated data are transmitted to more powerful remote resources. However, the network overhead of this scheme may not suffice for the real-time applications of VANETs. In a VANET scenario, numerous vehicles will rely on a single base station for requests and data upload. Forwarding large amounts of data to a single node would likely results in network congestion and would be exacerbated in areas of high traffic. One possible solution lies in the concept mobile edge-computing (MEC), which is a paradigm in which computational tasks distributed throughout the network and often kept closer to the end-user. Ref. [13] emphasizes the importance of NFV and software defined networking (SDN) in MEC and note that vehicular networks are a primary market driver. A method to optimize the usage of a single cloud resource is presented in [14] and could prove useful for groups of users that are in close proximity to a single edge resource. Expanding this method to MEC would allow users to be clustered based on their nearest resource and could provide optimal utilization of that resource. Two main goals of MEC are to reduce network congestion and minimize delay experienced by the user. By reducing the distance the data must travel over the network, executing tasks at an edge node minimizes the impact on the network. This also reduces delay as both the request and response will reach their destination sooner. While the reduction in delay for trivial requests will be negligible, situations involving transmission of data could see significant reductions. For computational tasks, these reductions in delay are also dependent on computational capability of each resource. For example, when delegating a task to a less powerful nearby resources; the reduction in delay may be overshadowed by an increased computational time of the local resource. The task of resource provisioning in a vehicular network would be complex and requires a sophisticated system to monitor resources and distribute tasks accordingly.

Vehicular networks present a unique opportunity for use in both MEC and what is known as fog computing (FC). Similar to MEC, FC seeks to perform computational tasks on the most logical resource so as to reduce the overall impact on the storage, computational, and network resources [15]. However, fog computing will employ resources even closer to the end-user, including the end node itself. Vehicular networks with appropriate infrastructure can utilize edge computing by off-loading some tasks to the RSUs, base stations, cell towers, or other infrastructure that provides connection to the network backbone. Additionally, vehicular networks can further leverage FC by employing powerful on-board computational hardware that is expected to reside on future vehicles. The inclusion of such hardware in the network creates a new architecture that could contextually execute, store, or transmit any data in the most logical and efficient fashion. The SDFC–VeNET architecture proposed in [16] employs a combination of both SDN and FC to provide an effective mechanism to minimize the delay associated with handover. Through rigorous simulation, the authors demonstrate the benefits of FC in vehicular networks and its ability to support a large number of vehicles in a dense network. An additional concept that has gained traction in vehicular networking is the use of named data networking (NDN). NDN is an extension of content-centric networking (CCN) and is based on the premise that the primary function of a network is data dissemination and retrieval. In [17] the proposed Navigo scheme separates content based on geographic location and is simulated with a music streaming application. With regard to task delegation, the geographic classification of data used by the authors could be applied to resource manager in an edge computing network. Given that the biggest factor in performance is often delay, the default resource for a region could be designated as the geographically closest resource.

In this paper, we extend our work originally presented in [18] and provide a more in-depth analysis of the proposed architecture and its application to real-world test cases. Additional experiments are conducted with LTE wireless communications and a mobile node to better replicate the environment of vehicular networks. In our architecture, we assume that computational tasks can be delegated to any available resource throughout the network. This would include remote cloud servers, edge nodes, and the mobile devices themselves, where edge nodes are defined as computational resources distributed throughout the network. These could be in the form of computationally-enabled base stations, computationally-enabled RSUs, or small cloud servers. In this aspect, our technique is not unlike traditional edge computing in that we seek to utilize computational resources at the network edge and alleviate the load on remote resources. However, our framework differs from others in the way we separate requests based on their context and maintain a default resource for time-critical tasks. Traditional edge computing seeks to minimize delay and delegate tasks to the nearest resource that can accommodate the job. However, the priority of request can vary and depends solely on the application for which the request was made. For example, a task to process data collected from on-board sensors about overall long-term vehicle performance, would be classified differently than a task to process data collected on real-time traffic status. Creating a distinction between task types ensures availability of sufficient resources for time-critical jobs as they arise and allows delay-tolerant jobs to be completed as resources become available.

## 2. CAMEVAN: Contextual Architecture for Mobile Edge-Computing in Vehicular Networks

**Figure 1:**
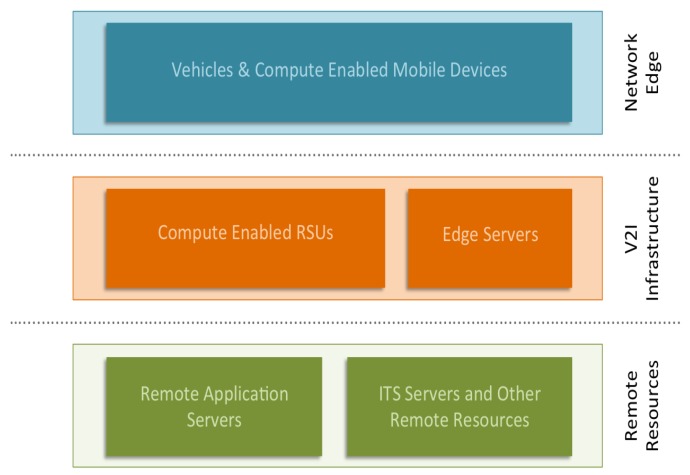
Contextual architecture for mobile edge-computing in vehicular networks (CAMEVAN) architecture.

In this work, we propose a contextual architecture for mobile edge-computing in vehicular networks (CAMEVAN) that seeks to delegate computational tasks in a more efficient way. Our architecture considers a variety of parameters when deciding where to send a request. Factors include: computational complexity, memory impact, data size, and delay. Real-time decisions are made for each job that take into account each factor and examine available resources to determine an optimal location for task completion. Our architecture works by handling computational tasks in real-time and making a decision on which available resources the task should be executed. It should be noted that our architecture is designed toward computationally intensive tasks and does not generate requests for data or other trivial requests to remote resources. The types of tasks we target are those that will have noticeable effect on CPU usage and network performance. These are tasks that wish to perform some type of data processing, or execute a computationally intensive task. In the case of tasks involving large amounts of data that needs to be processed, pre-processing or data-reduction can be delegated to the less powerful edge nodes to reduce the amount of data that must be transmitted to a more capable remote resource. Figure 1 shows an overview of the layers of our the CAMEVAN architecture. In the network edge layer, we assume that we have computationally-enabled edge devices that have the ability to perform modest computational tasks. In our architecture, we propose computationally-enabled RSUs (CERSUs) that act as the base station for V2I communication, but can also provide limited computational resources. In this layer, we also include edge servers, that contain computational resources closer to the network edge. The final layer is the remote resource layer, which represents any remote application server, web server, or ITS application server. The overall goal of the architecture is to minimize the distance any request or data must travel over the network. The delay for a computational request could be reduced if the edge resource possesses reasonable processing power and is geographically closer to the requesting node. Given that edge resources will have limited capabilities and be required to support a large number of users, we must define a method of ranking all available resources in real-time. Any benefit in reduced communication delay would be irrelevant if the edge resource is near full-utilization and a distant resource is idle. To measure the real-time utilization of computational resources, our architecture queries available resources and simultaneously measures the round-trip communication delay between the requesting node and resource. Given the recent enough statistics, we can then rank each resource and provide a recommendation for a given task.

**Figure 2:**
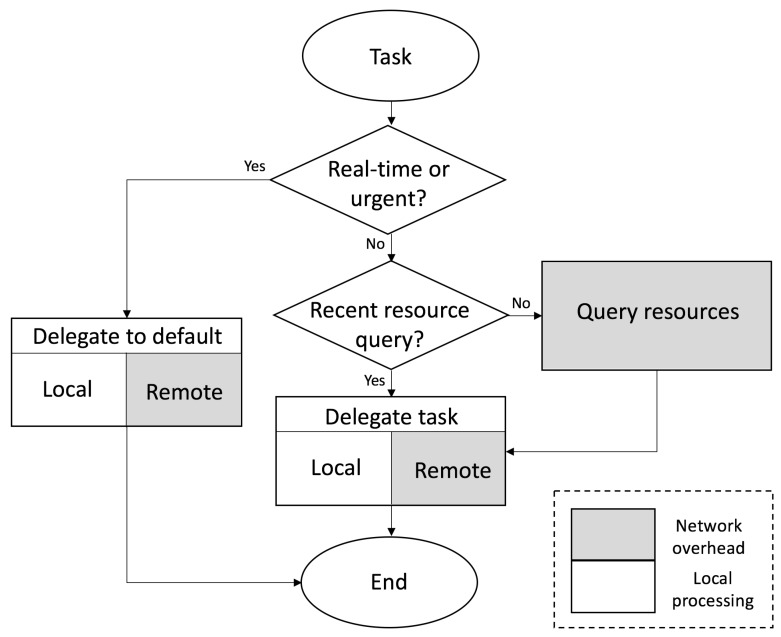
Design of CAMEVAN task manager.

In the CAMEVAN architecture, the task manager shown in Figure 2 is responsible for querying available resources and delegating tasks. Depending on the network configuration and type of computational resources available, the task manager could reside on each computational node or at end nodes only. For our case, we assume that the task manager exists at the end nodes of the network and delegates tasks to local and remote resources. Local resources are computationally-enabled edge nodes while remote resources are geographically distant servers. The task manager takes into account priority of the task and expedites execution when the given request is urgent or in support of a real-time application. These requests are delegated to default resources that can be both local and remote depending on the task complexity. Less time-critical tasks, along with those involving larger amounts of data are delegated based on a status query of available resources. Results from recent queries can be saved and used for subsequent requests with a threshold defining the lifetime of a query result. If a query is not recent enough, a new query is initiated and the results are saved to a resource table. The time required for a new query of all resources is equal to the highest round trip delay observed for all resources, frequent queries may create additional delay. In our experiments the observed round trip delay was on the order of a couple hundred milliseconds for a geographically diverse test-bed of resources. Increasing the lifetime of query table results could reduce latency and network overhead when during task delegation. However, if this threshold is too high the status of available resources may not be accurately reflected and values may become stale. In either case the task manager will delegate a given task to the best available resource based on real-time metrics.

In our architecture, V2I infrastructure and remote resource layers are not like those found in existing MEC architectures. Our architecture varies from others by seeking to delegate computational tasks to the most logical resource in a contextual way. The decision-making process is handled by a multiple-criteria decision analysis (MCDA) method, which takes into account both beneficial and non-beneficial criteria. The MCDA method we employ is the technique for order of preference by similarity to ideal solution (TOPSIS) [19], that works by assuming that the chosen alternative should have the shortest geometrical distance from the positive ideal solution (PIS) and the farthest distance from the negative ideal solution (NIS). TOPSIS assumes that each criterion should be maximized or minimized. In our case, we have a mixture of both beneficial and non-beneficial criteria. The alternatives are the different resources available for task delegation. TOPSIS provides a ranking of these alternatives, which ranks our resources from best to worst with respect to the parameters of the individual computational task. For example, a task with a high memory requirement may be delegated to the resource with the most free memory. One the other hand a task with a small memory requirement can be delegated to a resource with available processors but little free memory.

The first step of TOPSIS is to create a decision matrix (DM). For our application, the decision criteria were parameters such as computational complexity, memory impact, data size, and delay which we refer to as $C_{1},C_{2},\ldots ,C_{n}$. Our alternatives use different computational resources which we refer to as $A_{1},A_{2},\ldots ,A_{n}$. The elements of the matrix correspond to the values of criteria *i* with respect to alternative *j*.

$$\begin{array}{ccccc} & & & \begin{array}{cccc} C_{1,n} & C_{1,2} & \cdots & C_{1,n} \end{array} & \\ DM= & \begin{array}{c} A_{1,1}\\ A_{2,1}\\ \vdots \\ A_{m,1} \end{array} & ( & \begin{array}{cccc} x_{1,1} & x_{1,2} & \cdots & x_{1,n}\\ x_{2,1} & x_{2,2} & \cdots & x_{2,n}\\ \vdots & \vdots & \ddots & \vdots \\ x_{m,2} & \cdots & \cdots & x_{m,n} \end{array} & ) \end{array}.$$

Next, we create a normalized decision matrix (NDM) *R* from the DM, $x_{ij}\left(mn\right)$. $R=(r_{ij})mn$ where the normalization method is defined as:

$$r_{ij}=\frac{x_{ij}}{\sqrt{\sum_{k=1}^{m}X_{kj}^{2}}},i=1,2,\ldots ,m,j=1,2,\ldots ,n.$$

We then determine the weighted decision matrix *t* with:

$$t_{ij}=r_{ij}\cdot w_{j},i=1,2,\cdots ,m,j=1,2,\cdots ,n,$$

where

$$w_{j}=\frac{W_{j}}{\sum_{k=1}^{n}W_{k}},j=1,2,\cdots ,n.$$

We can then find the PIS and NIS. PIS as the best alternative $A_{b}$ and NIS as the worst alternative $A_{w}$ are found with the following:

$$A_{w}=\{\langle max\left(t_{ij}\right)|i=1,2,\cdots ,m|j\in J_{-}\rangle ,$$

$$\langle min\left(t_{ij}|i=1,2,\cdots ,m\right)|j\in J_{+})\rangle \}\equiv t_{wj}|j=1,2,\cdots ,n.$$

$$A_{b}=\{\langle min\left(t_{ij}\right)|i=1,2,\cdots ,m|j\in J_{-}\rangle ,$$

$$\langle max\left(t_{ij}|i=1,2,\cdots ,m\right)|j\in J_{+})\rangle \}\equiv t_{wj}|j=1,2,\cdots ,n,$$

where $J_{+}$ and $J_{-}$ represent beneficial and non-beneficial criteria respectively, and are defined as follows:

$$J_{+}=\left\{j=1,2,\cdots ,n|j\right\}.$$

$$J_{-}=\left\{j=1,2,\cdots ,n+j\right\}.$$

L2-distance between alternative *i* and the worst condition $A_{w}$ is calculated with:

$$d_{iw}=\sqrt{\sum_{j=1}^{n}{\left(t_{ij}-t_{wj}\right)}^{2}},i=1,2,\cdots ,m.$$

Similarly, the distance between alternative *i* and the best condition $A_{b}$ can be found with:

$$d_{ib}=\sqrt{\sum_{j=1}^{n}{\left(t_{ij}-t_{bj}\right)}^{2}},i=1,2,\cdots ,m,$$

where $d_{iw}$ and $d_{ib}$ are the Euclidean distances between the target alternative *i* and the worst and best conditions.

Finally, we calculate the similarity to the worst condition with:

$$s_{i}w=\frac{d_{iw}}{\left(d_{iw}+d_{ib}\right)},0\leq s_{iw}\leq 1,i=1,2,\cdots ,m.$$

The value $s_{iw}=1$ if and only if the alternative has the best solution and $s_{iw}=0$ if and only if the alternative has the worst condition. $s_{iw}$ can then be used to rank the alternatives as $s_{iw}\left(i=1,2,\cdots ,m\right)$.

Our architecture makes use of these rankings to choose an optimal resource for execution of each task. Adjusting the weights of each given criteria can bias the system to be sensitive to a certain criteria. In our case, we have both beneficial and non-beneficial criteria, meaning that some values are better when high and some are better if lowered. For instance, one of our criteria is the delay which is defined as the round-trip ping time that is affected by geographical distance and represents network overhead. In time-sensitive situations, delay criteria would have a higher weight to ensure that important jobs are executed in a timely manner. Conversely, jobs with delay-tolerance such as data off-loading, could be given a lower weight for delay-tolerance, since the task is not time-sensitive. Adjusting the weights of criteria for different job types ensures that resources are used efficiently for a range of situations.

## 3. Comparison of Task Delegation Scheme with Experimental Results

To demonstrate the trade-offs of executing computational tasks in different geographical regions, we developed two test cases that performed computations on data sets of varying sizes. The data we focus on for these tasks is GPS trace data that was collected by an Android smart phone while in a vehicle under normal driving conditions. The data was retrieved from a Google Maps Timeline and was filtered to include only records collected when the device was predicted to be in a moving vehicle. The first of the two tasks was to parse the raw GPS trace data, which is in the JavaScript object notation (JSON) format, to a more condensed and readable comma separated values (CSV) format. This task involves a large amount of text parsing, whereas the second task involves a large number of geographical distance calculations using a Haversine-based formula [20]. The second task seeks to discover which intersections were encountered by the driver over a period of time. This task requires a database of intersection coordinates, which is obtained through OpenStreetMap and the Overpass-Turbo API. Our data was collected in the Washington, D.C. metro area and spans the period of June 6 2018 to August 6 2018. We break this data set into chunks representing one week of GPS data each. It should be noted that the size of each chunk can vary slightly depending on the amount of driving. On average each week of raw data consisted of 2639 GPS coordinate pairs.

For our remote servers, we utilized the Google Cloud Infrastructure, particularly Google Cloud Compute Engine. This service allowed us to easily replicate the server in different geographical regions and adjust the hardware of each resource. In each case our servers were configured identically with Ubuntu 14.04 and all processing is handled by PHP, Java, and Apache web server. For portability, each task was implemented in Java and exported to a Jar file. Requests to the server were handled by a simple PHP script that makes calls to execute the correct Jar file with the corresponding data.

### 3.1. JSON Parsing

**Figure 3:**
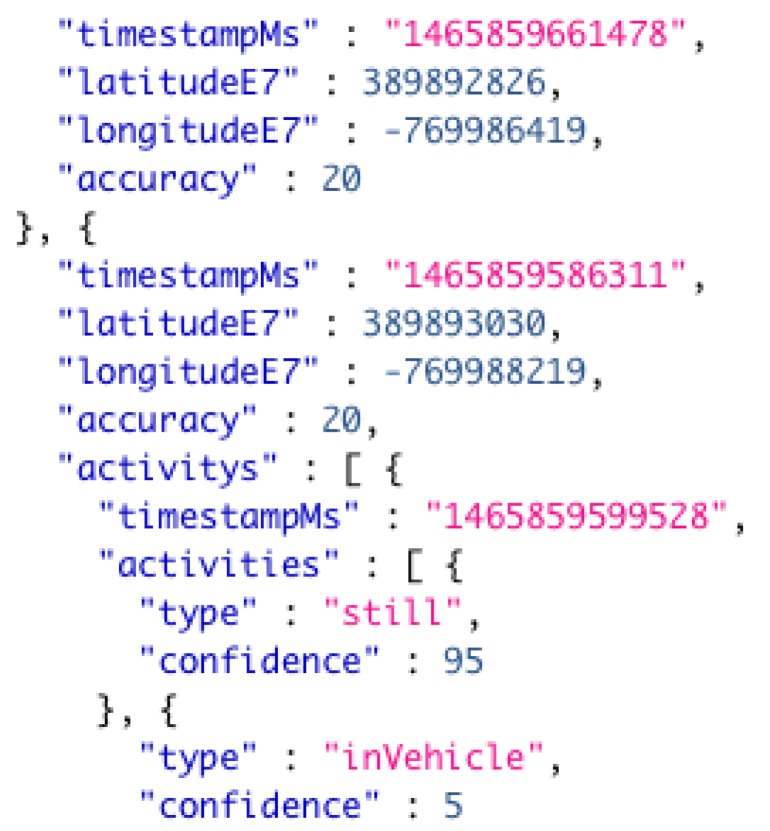
Example of raw data in original JavaScript object notation (JSON) format.

**Figure 4:**
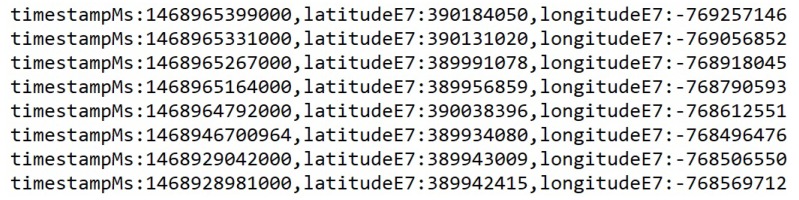
Example of parsed data in comma separated values (CSV) format.

Our first computational task was to parse the raw GPS trace data from a JSON format to CSV format. This task involved simple text parsing in which the elements of JSON notation were removed and the desired attributes were left in a CSV format. We extracted its attributes from the raw data including: time stamps, GPS coordinates, and observed activity type at the given time stamp. The task was implemented in Java programming language and made use of a series of regular expressions to extract the target attributes. Once the records were reduced to the desired attributes, we then further reduced the data by taking only records with an observed activity type of driving or “inVehicle”. Figure 3 shows an example record in the raw JSON format, and Figure 4 provides an example of the final CSV output after the parsing process.

The raw data was continually collected and contained records with various activity types such as “still”, “inVehicle”, “onFoot”, “onBicycle”, and so on. Since we only targeted the records that were collected while the user was driving or “inVehicle”, our parsing process resulted in a significant reduction in the size of the data set. For example, one week of GPS trace data in the raw JSON format is approximately 27,841 lines of text and has a size of 657 KB. Parsed to CSV format without removing the undesired records, the data is approximately 2283 lines of text and 369 KB. Once we have extracted all activity types other than “inVehicle”, the data was approximately 109 lines of text and 36 KB. This reduction in data size greatly reduced the complexity of any future processing. In our test cases, this parsed data was the direct input to our next task. This process also demonstrated value in an edge computing system in which we may decide to perform trivial tasks at the edge of the network such as data pre-processing or reduction. In this case, performing the parsing task at the network edge can greatly reduce latency and communications overhead. Compression of the data would serve to further reduce the amount of data to be transmitted. However, for our test cases the data sets were small and compression was not necessary.

### 3.2. Intersection Discovery

**Figure 5:**
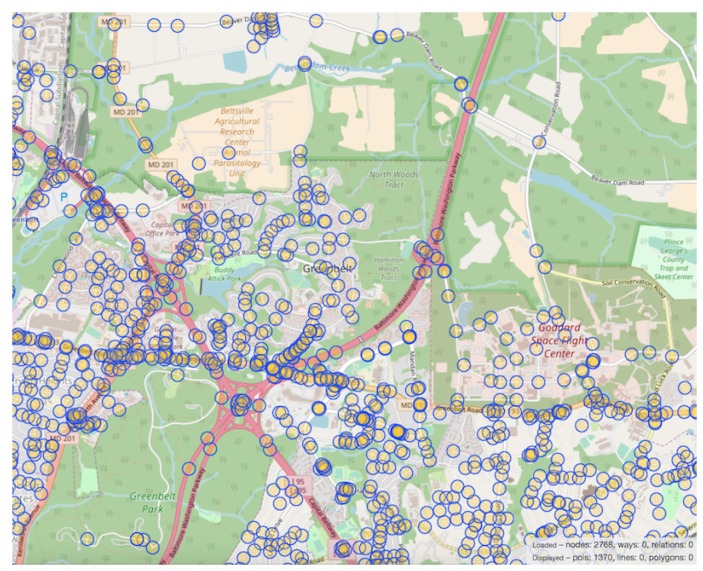
Intersection geo-location.

Another task we employed to evaluate our architecture is the discovery of which intersections were encountered by the driver. We accomplished this by creating a database of all intersections in the region in which the vehicle operated. The coordinates of each intersection were found with OpenStreetMap and simple script that leverages the Overpass-turbo API. Figure 5 shows an example of the method we used to retrieve the coordinates of intersections, along with a graphical view of markers denoting intersections in a portion of our target area. The first step in discovering intersection encounters was to define the distance *r* from an intersection that the user must be within for a particular intersection to be considered visited. The parameter *r* represents the radius of a circle centered around the geographical coordinates of an intersection. If a vehicle passes in this area, the intersection was said to have been visited. As previously mentioned, we utilized a Haversine-based formula that takes into account the spherical shape of Earth when measuring distance between two locations. Haversine assumes a perfect sphere, so we use Earth’s equatorial radius of 6378 km. The distance *d* between the vehicle coordinates and the intersection can then be found as:

$$d=2e\arcsin\left(\sqrt{\sin^{2}\left(\frac{\phi_{2}-\phi_{1}}{2}\right)+\cos\left(\phi_{1}\right)\cos\left(\phi_{2}\right)\sin^{2}\left(\frac{\lambda_{2}-\lambda_{1}}{2}\right)}\right),$$

where $\phi_{1}$, $\phi_{2}$ are the latitudes of the intersection and the vehicle location, $\lambda_{1}$, $\lambda_{2}$ are longitudes, and *e* is the radius of Earth.

### 3.3. Experimental Results

In this section, we describe a test scenario using real-world data and the two computational tasks previously defined. We demonstrate the use of a decision-making process using the TOPSIS method described in Section 3 Our test cases consider three criteria and five alternatives during the decision-making process. The criteria considered include: available processing resources, memory available, and delay. Our alternatives were computational resources at different geographical locations. Identifiers and the locations represented are defined as follows: central: Council Bluffs, Iowa USA; west: the Dalles, Oregon USA; east: Ashburn, Virginia USA; cin: Cincinnati, Ohio USA; and local: requesting device. At each location, we duplicated the computational server and create virtual machines with identical CPU and memory resources. We also adjusted the resources of the servers, giving each instance more processing power or more memory. In comparisons of regions, we always created instances with identical, or as identical as possible, resources to avoid any differences arising from imbalanced hardware. The local resources were configured as closely as possible to the other resources and are used to represent the edge nodes on the network which are the vehicles in our application.

#### 3.3.1. CAMEVAN Example with the TOPSIS Decision-Making Method

To demonstrate how decision-making is handled in our architecture, we work through an example using various test cases. Recall that the TOPSIS method requires a decision matrix DM. To create this matrix, we label the columns with our criteria and the rows with our alternatives. Non-normalized attribute values along with the weights assigned to each criteria, are shown in Table 1.

The “cpu” criteria represents the real-time processor load at the corresponding alternative and is represented as a percentage of current processor usage. The “mem” denotes the amount of free memory available in Gigabytes and delay is an indicator of the round-trip communication delay in milliseconds. In each alternative, the computational resources are kept identical with the exception of the “cin” alternative. In the case of the “cin” alternative, we were limited in options for available infrastructure. The only notable difference between the “cin” node and the others is a reduced amount of memory. The “cin” node was limited to 4 GB of memory while others were allocated 15 GB, which was due to physical hardware limitations. At any rate, this difference in memory does not adversely affect any testing, as no test case required over 2 GB of memory. However, it may make this asset less-favorable in the TOPSIS ranking due to lower amount of memory available. Each virtual server is configured with a four-core Intel Xeon processor. Additional testing was conducted with singe and dual core configurations, but the best performance was observed with the four cores. Thus, run-time results presented were obtained with the four-core configuration. Configurations with fewer processing cores showed very similar results in terms of ranking and had only slightly longer run times.

Before creating a ranking of our alternatives, we must transform the decision matrix into a normalized-weighted decision matrix (NWDM). Recall that the normalized decision matrix *R* is found as:

$$r_{ij}=\frac{x_{ij}}{\sqrt{\sum_{k=1}^{m}x_{kj}^{2}}},i=1,2,\ldots ,m,j=1,2,\ldots ,n$$

NWDM was found by multiplying each attribute in *R* with it’s respective weight. Applying this to the matrix values in Table 1, we obtain the NWDM shown in the first four columns of Table 2. From this matrix we can easily find the PIS $A_{w}$ and NIS $A_{b}$ with the formulas previously defined. From the NWDM we can see that:

$$A_{b}=\left\{0.0483412,0.132433,0.0\right\}$$

$$A_{w}=\left\{0.23687188,0.02417878,0.23759193\right\}.$$

With these values known, we can then calculate the separation distance of each alternative to the best and worst solution. Finally we measured the closeness of each alternative to the ideal solution and rank each them accordingly. The rankings obtained for each alternative are shown in last column of Table 2.

#### 3.3.2. Comparison of TOPSIS Ranking with Experimental Results

To validate our approach, we implemented two test cases discussed previously and conducted a series of trials on the alternative infrastructure defined in the TOPSIS example. Recall that servers are located in different geographical locations to represent the communication delay associated with remote servers. Testing is accomplished by sending processing requests to each remote resource from a local lab machine connected to a WiFi network. We chose a wireless network to better replicate vehicular networks. However, a stationary lab machine does not accurately emulate the environment of a vehicular network. To remedy this shortcoming, we conducted another series of trials in which the requests were sent to the remote resources over an LTE network, as Cellular-V2X communication has become more popular. However, experiments were conducted with an off-the-shelf smart phone that operates in the LTE frequency band ranging 700 MHz to 2500 MHz. In the realm of vehicular networks, LTE is being considered as an enabling technology for vehicular operating in the 5.9 GHz frequency band as LTE-V2X [21]. Given that these technologies are still in development and an LTE connected vehicle was not available for experimentation, the best configuration for our experiments was an LTE smart phone.

**Figure 6:**
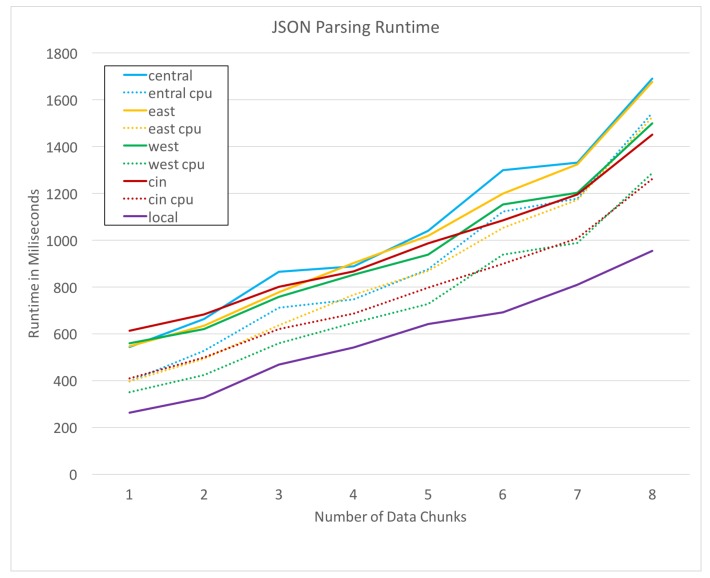
Run-times of JSON parsing for each resource.

**Figure 7:**
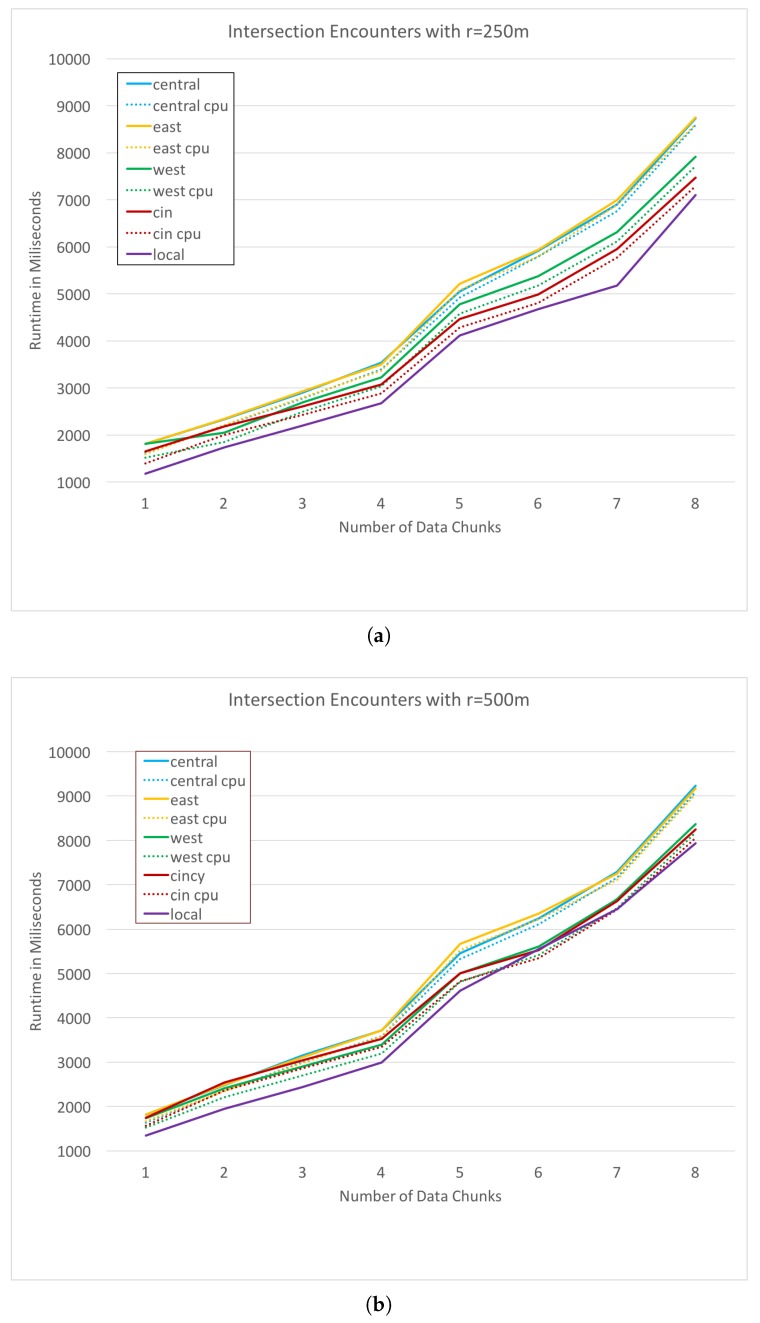
Intersection encounter analysis runtime with r=250 m (**a**) and r=500 m (**b**).

Our first test case is JSON parsing, as previously described in this section. Figure 6 plots the run times of the JSON parsing process on each remote resource for a range of data set sizes. The horizontal axis specifies the amount of data by the number of weeks over which it was collected. For example, the points at *x*-axis value 1 represents the run-time of JSON parsing for 1 week of GPS trace data. In both Figure 6 and Figure 7, dotted lines represent the cpu time taken for the process, while the solid lines represent the total run time in milliseconds (ms) for the request including cpu time and network overhead. Our second test case is an intersection encounter discovery, as defined previously. This task is much more cpu intensive, as it requires a large number of Haversine distance calculations. Figure 7a,b plot the cpu and total time for each alternative with an intersection search radius values *r* of 250 and 500 m respectively.

With the experimental results plotted for both processes, we can now compare the ranking provided in our TOPSIS model. If our ranking is accurate, we should see similarities between the performance of the remote resources and the rankings we obtained with the TOPSIS method. Comparisons between TOPSIS rankings and the actual performance are shown in Table 3. Our alternatives or server resources are compared by observing the total time for each processing task to complete. In the case of the JSON parsing task we can see, in Table 4, that ranking of our experiments vary from the TOPSIS ranking in two of five cases. However, on closer inspection, we find that the two incorrectly ranked cases are reversed. Upon inspecting the average run time across all test cases for these two alternatives, shown in Table 4, we find that $A_{east}$ with an average run time of 1010.125 ms and $A_{west}$ with an average run time of 948.125 ms, only differ by 62 ms overall. Similarly, the rankings for the intersection encounter discover task exhibits a similar property in that two of five ranks are incorrect and are reversed from what TOPSIS provides. In this case, the difference between the two cases is even smaller than that of the JSON rankings. The average run times for the two incorrectly ranked alternatives $A_{central}$ and $A_{east}$ are 4923.75 ms and 4947.875 ms respectively and differ, on average, 24.125 ms across all test cases. These results demonstrate that a MCDA method, TOPSIS in our case, can effectively make decisions about which resources are best for task execution. In a real-world system, cpu loads, memory availability, and delay vary from the numbers we observed due to the higher system loads and higher number of users. In each case, our systems consisted of idle servers with only a single user making requests. However, the parameters we use are relevant to real-world scenarios and should provide similar results. It is to be noted that the weights for each of our criteria could easily be adjusted to bias the decision-making toward a given attribute.

## 4. Further Analysis of Delay

In a challenging environment such as a vehicular network serving autonomous vehicles, delay can have a significant impact on real-time applications. Thus, an architecture that has some a priori knowledge of network delay, and the ability to make informed decisions on which resource to send a request, is desirable. To evaluate the variations in communication delay, we conduct a serious of experiments and record the time spent processing the request along with the time taken for the requesting node to receive the response.

**Figure 8:**
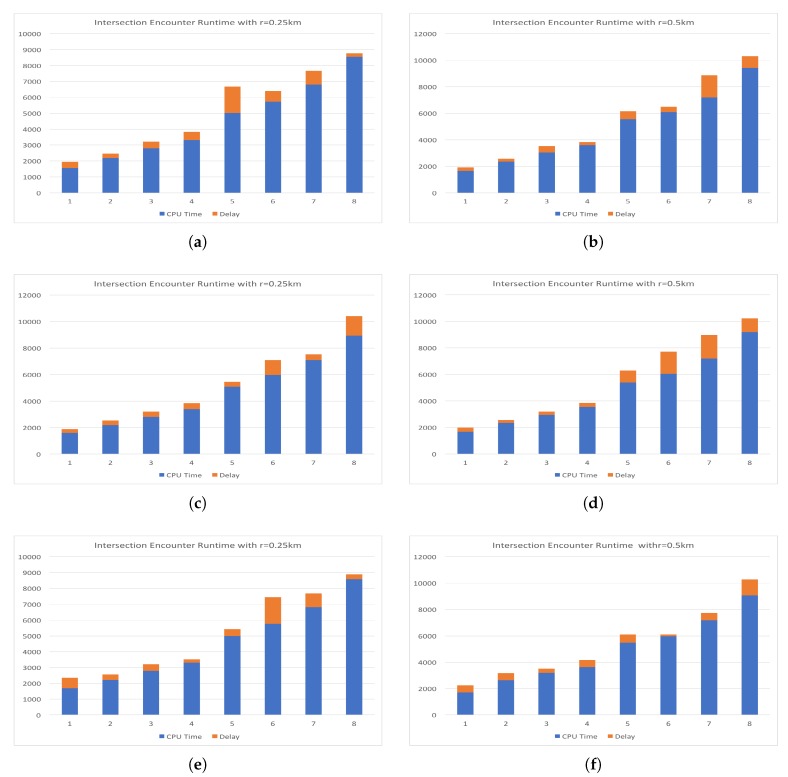

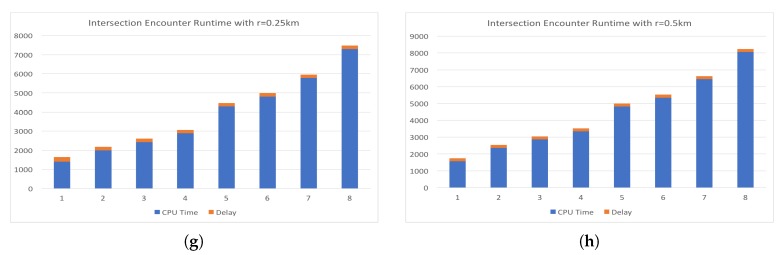
Comparison of CPU time and round trip communication delay for geographically dispersed resources. The sum of the bar segments is equal to the total time for the request to be sent, processed, and results returned. Figures (**a**) and (**b**) illustrate the run time and delay for requests sent to the “east” server, (**c**) and (**d**) the “central”, (**e**) and (**f**) the “west”, and finally (**g**) and (**h**) the “cin” server.

In Figure 8, we target the three geographically dispersed server locations and the local server for delay analysis. Each server is sent a series of requests with increasing data size, where data is split into chunks by week. The number of data chunks for each test are shown on the plots as the numbers 1 though 8 on the *x*-axis. The total time of the requests is shown on the *y*-axis in milliseconds. The overall time taken for each request is the sum of the two bar segments. CPU time is shown in blue and delay in orange. Recall that the “cin” server was a resource allocated in our lab at the University of Cincinnati and had the lowest delay since it was very close to the requesting node. The request made to the servers is the intersection discovery technique detailed in a previous section of this paper. Here we have passed two large values for *r* of 0.25 km and 0.5 km. Here the intersection coordinates could be derived by taking the centroid of the points that are found to be in the circle with radius *r*. The run time increased in a linear fashion and was of little interest for our purposes. However, it can easily be seen that the delay varies greatly between requests. The spread of this delay can be used to provide a better representation of what delay can be expected for a specific resource. While the round trip communication delay may have improved with a wired connection, we purposefully chose a wireless standard since we are seeking to emulate a wireless environment. Each of the experiments in Figure 8 has been conducted on a lab machine and requests were sent with a WiFi connection to the Internet. Since our target environment involves mobile nodes and wireless communication, we conducted additional experiments where requests were sent over wireless LTE network in various locations and while the requesting node is moving. The LTE network provider for the experiments was Google Fi (known as Project Fi at the time of the experiments) and the experiments are conducted with a Google Pixel 2 XL device. Experiments with the mobile device were conducted while the device was moving at a walking pace in a congested area. The path covered in the mobile experiments traversed the campus of the University of Cincinnati, Cincinnati, OH, USA, which has many trees, tall buildings, and other obstructions.

**Figure 9:**
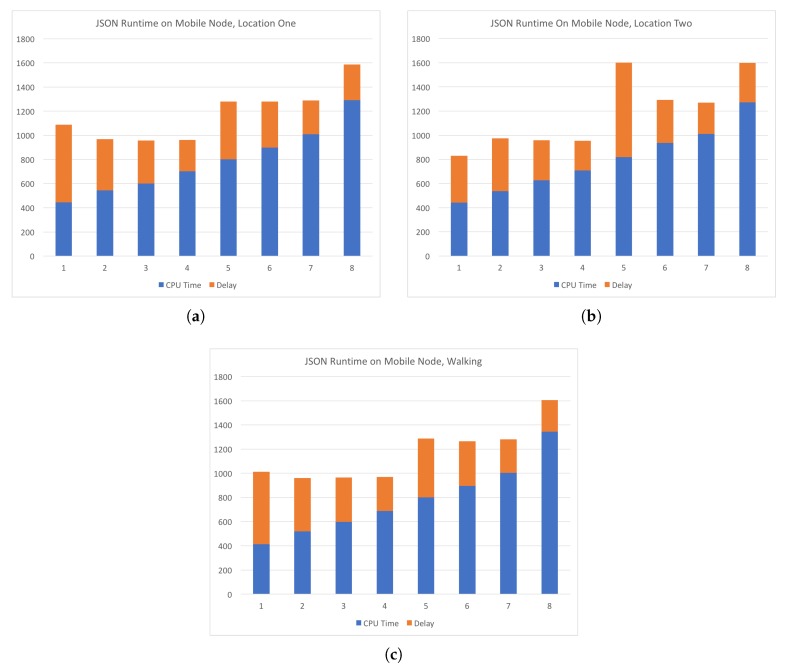
Comparison of CPU time and delay for requests made from a mobile device to the “central” server location. (**a**) and (**b**) are requests made while stationary at two locations and the results in (**c**) were obtained while walking between the two locations.

**Figure 10:**
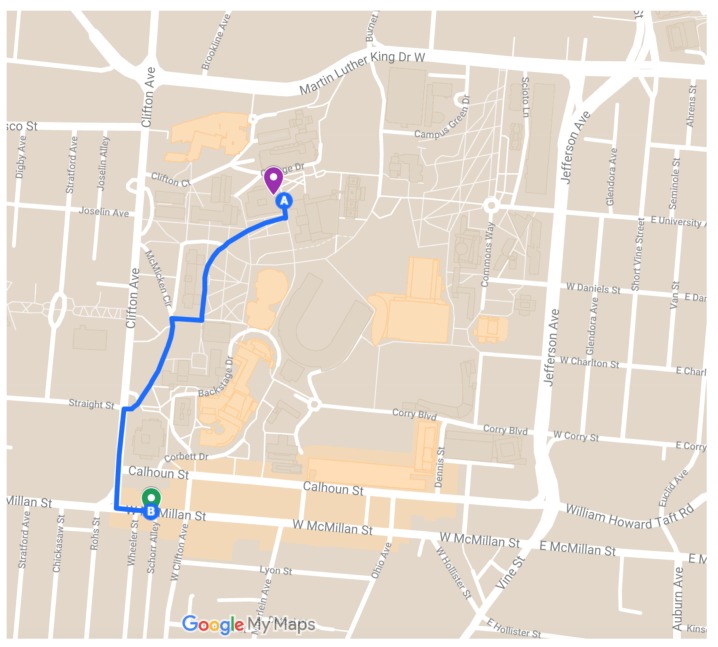
Locations used in mobile node experiments and the path walked between them for experiments conducted while the device was moving.

**Figure 11:**
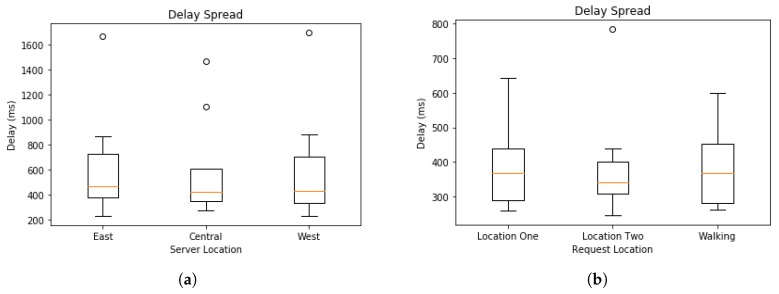
Analysis of delay spread with high fliers shown. (**a**) Analysis of delay spread for intersection encounter requests sent to the “east”, “central”, and “west” servers. (**b**) Analysis of delay spread from a mobile device with requests being made while stationary at locations one and two and while walking between the two locations.

Figure 9 shows experiments in which requests were sent to the central server from two locations and while the device is moving at a walking pace between the two locations. The computations requested were the JSON parsing task that has been detailed in an earlier section. Subsequent requests had increasing amounts of data to be processed. The number of data chunks is shown on *x*-axis and the total time in milliseconds is shown on *y*-axis. As in Figure 8, CPU time is shown in blue and delay in orange. The two locations and the path walked between them are shown in Figure 10. Location one is shown as the magenta marker and location two is shown as the green marker and the path walked between them as the blue line. Location One has coordinates (84.51734,39.13329) and Location Two has coordinates (−84.52003,39.12781). In Figure 9, we see that the delay on average is more consistent than that of the experiments conducted using a WiFi connection to the Internet. However, additional measurements at the time of the experiments have shown inclination as to why this is the case. Network traffic at the time could have been higher than normal or some other unknown circumstance could have had some effect on the network performance. In any case, our experiments show that on average the delay of the LTE network had slightly less delay than the WiFi connection. A clearer comparison of the delay across all experiments is shown in Figure 11, which plots delay in milliseconds on the *y*-axis. Figure 11a illustrates the spread of the delay from the experiments shown in Figure 8. The “cin” server was excluded form this analysis since it is very close to the requesting node and had very small delay in all test cases and had a minimum of 176, maximum of 249, and average of 185.0625 milliseconds. The delay spread for the mobile experiments are shown in Figure 11b. In both spread figures, the high fliers are shown with circles. In Figure 11b, we see that the delay at location one had high spikes in delay compared to the delay of location two. We attribute this to the location being inside a large multistory building and assume the structure decreased signal strength. Additional tests would be required to verify that this is the case.

## 5. Conclusions and Future Work

In this paper, we introduced the CAMEVAN architecture for vehicular networks that supports edge-computing and employs a multiple-criteria decision analysis method to choose an optimal resource for delegation of computational tasks. We present an example using the TOPSIS decision-making method, and validate our results with experiments on geographically diverse test bed of computational resources. Our experimental results support the results of the decision-making process and is accurate for the majority of test cases. Our testing considered multiple criteria in the decision-making process, including real-time cpu load, real time memory availability, and real time network path delay. To produce a more robust decision-making model, more criteria could be considered or criteria weights could be adjusted to better suite individual situations.

In our decision-making process, we consider criteria that pertain to the computational resources and their geographical location. In future work, we would like to incorporate criteria that relate to an individual job, such as the amount of data that must be transmitted to the computational resource, computational complexity of the task, and priority of the task. Incorporating these criteria that vary for each job, would allow decisions to be made with respect to both the computational resources available and characteristics on the computational job. This type of decision-making process, would allow the model to bias jobs that require more or less of a specific resource to be the best alternative. For example, if a job is computationally intensive, but requires very little memory, alternatives with small amounts of available memory wouldn’t be considered less favorable then alternatives with large amounts of available memory.

For future work, we would like to continue the experiments with a mobile node and incorporate more parameters into the allocation process. Mobile nodes present a unique challenge in that they have significantly varying network delay. Accommodating this uncertainty would require a robust and intelligent system. One potential approach would be to maintain historical measures of average delay for each resource. With each request the delay could be measured and used to update the information about a specific resource. Our experiments with delay have shown that geographically diverse resource can have differing delays and could be subjected to unexpected spikes in delay. We have also shown that current wireless communication technologies exhibit consistent delay while travelling at low speeds. While the average delay is higher than an ideal level for geographically distant resources, mobile edge computing would move resources closer to the edge, thus reducing the delay while maintaining consistency. Finally, it would be beneficial to repeat the experiments for a mobile device in a moving vehicle or, if possible, from an Internet connected vehicle.

## Figures and Tables

**Table 1:** Decision matrix with attribute values.

	Criteria		
Alternatives	cpu (w=0.35)	mem (w=0.25)	delay (w=0.4)
$A_{central}$	6.54%	14,679 MB	151.375 ms
$A_{east}$	2.37%	14,481 MB	145.75 ms
$A_{west}$	2.31%	14,492 MB	207.875 ms
$A_{cin}$	1.4%	2680 MB	187.375 ms
$A_{local}$	6.86%	11,202 MB	0 ms

**Table 2:** Normalized-weighted decision matrix (NWDM) with ranks.

Criteria’s				
Alternatives	cpu (w=0.35)	mem (w=0.25)	delay (w=0.4)	Rank
$A_{central}$	0.225822	0.132433	0.173015	5
$A_{east}$	0.0818347	0.130647	0.166586	2
$A_{west}$	0.079763	0.130746	0.237592	4
$A_{cin}$	0.0483412	0.0241788	0.214161	3
$A_{local}$	0.236872	0.101064	0.0	1

**Table 3:** Comparison of technique for order of preference by similarity to ideal solution (TOPSIS) ranking with test cases.

Alternatives	TOPSIS Rank	JSON Rank	Int. Enc. Discovery Rank
$A_{central}$	5	5	4
$A_{east}$	2	4	5
$A_{west}$	4	2	2
$A_{cin}$	3	3	3
$A_{local}$	1	1	1

**Table 4:** Average run-times in milliseconds (ms).

Alternatives	JSON (ms)	Int. Enc. Discovery (ms)
$A_{central}$	1040.5	4923.75
$A_{east}$	1010.125	4947.875
$A_{west}$	948.125	4513.375
$A_{cin}$	960.25	4532
$A_{local}$	587	4160
